# Impact of Coronavirus Disease 2019 on Cardiac Arrhythmia Care: Experience of a Spanish Tertiary Hospital During the Health Crisis Triggered by the First Wave of the Pandemic

**DOI:** 10.19102/icrm.2021.120903

**Published:** 2021-09-15

**Authors:** Adolfo Fontenla, Daniel Rodríguez-Muñoz, Luis Borrego-Bernabé, Isabel Montilla-Padilla, Álvaro Marco del Castillo, Javier Ramos, Ana Isabel Fernández-Arranz, María López-Gil, Fernando Arribas, Rafael Salguero-Bodes

**Affiliations:** ^1^Cardiology Department, Hospital Universitario 12 de Octubre, Madrid, Spain; ^2^Research Institute Hospital Universitario 12 de Octubre (i + 12), Madrid, Spain; ^3^Centro de Investigación Biomédica en Red de Enfermedades Cardiovasculares (CIBERCV), Carlos III Health Institute, Madrid, Spain; ^4^Medicine Department, Faculty of Medicine, Complutense University of Madrid, Madrid, Spain

**Keywords:** Arrhythmia unit, cardiac electrophysiology, coronavirus, COVID-19, health management

## Abstract

The coronavirus disease 2019 (COVID-19) pandemic has resulted in a deep restructuring of cardiovascular care, especially in the setting of cardiac arrhythmia units, which are characterized by a wide variety of clinical and interventional activities. We describe the experience of a large university hospital deeply hit during the COVID-19 health crisis (first outbreak of the pandemic), focusing on the exceptional measures implemented and their impact in terms of outcomes. We performed a retrospective study comparing the human and structural resources and the activity of a cardiac arrhythmia unit in a Spanish tertiary hospital for two consecutive periods: from January 12, 2020, to March 8, 2020 (“pre-COVID stage”), and from March 9, 2020, to May 2, 2020 (“COVID stage”). Data were contextualized within the number of confirmed COVID-19 cases in the region of Madrid. The measures implemented were promotion of non–face-to-face consultations, selection of urgent procedures, design of a “COVID-free” circuit for outpatient interventions, and protocolization for patients with COVID-19. A total of 3,526 consultations and 362 procedures were performed. During the COVID stage, the number of consultations remained stable, and the electrophysiology rooms’ activity decreased by 55.2% with a relative increase in the number of urgent-hospitalized cases attended (11.8% COVID-19-positive patients). The electrophysiology rooms’ activity returned to “normal” in the last week of the COVID stage, with no contagion being detected among patients or professionals. In conclusion, the measures implemented allowed us to respond safely and efficiently to the health care needs of patients with arrhythmias during the COVID-19 crisis and may be useful for other institutions facing similar situations.

## Introduction

During the last two years, the novel severe acute respiratory syndrome coronavirus 2, has spread worldwide, triggering a health crisis unprecedented in recent history. Transmitted through respiratory droplets, this virus is capable of causing coronavirus disease 2019 (COVID-19), sometimes producing severe and even fatal symptoms.^1^ Old age, hypertension, obesity, diabetes, and cardiovascular diseases are risk factors for severe COVID-19,^2^ affecting the majority of patients seen in cardiology departments.

As a consequence of the first COVID-19 outbreak in Spain, there was a deep structural and functional reorganization at all health care levels with the aim of increasing the system’s capacity during the period of highest demand for caring for COVID-19 patient care. All non-urgent activity of clinics, diagnostic tests, daytime hospitalization, scheduled admissions, and surgical interventions were suspended, directly impacting the organization of the arrhythmia units (as well as other intervention-based health care services). Consequently, adapting the activity of these units to the new scenario poses a challenge due to the high prevalence, mortality, and disability rates caused by cardiac arrhythmias and the need to address rhythm disorders in hospitalized patients with COVID-19.

The aim of this paper was to describe the impact of the COVID-19 epidemic on the clinical management of the arrhythmia unit of one of the most deeply hit tertiary hospitals during the health care crisis that took place in March and April of 2020 in Madrid, the extraordinary measures implemented, and their performance in terms of outcomes.

## Methods

Hospital Universitario 12 de Octubre is a large third-level public university hospital in the Community of Madrid, which serves a population of around 500,000 inhabitants and has a usual inpatient census of 1,050. A retrospective descriptive and observational study was carried out where all data on health personnel management, clinics, and electrophysiology rooms dependent on the arrhythmia unit of Hospital Universitario 12 de Octubre (AUH12O) were collected during the 16 weeks between January 12 and May 2, 2020.

The AUH12O attends two types of outpatient clinics: medical and nursing clinics. Prior to the pandemic, four medical clinics a week (three arrhythmia clinics and one general cardiology) were attended in person. Additionally, there were nine clinics a week for follow-up of cardiac devices (pacemakers, defibrillators, and implantable Holter monitors), attended by nurses in person or in a virtual format (using remote monitoring systems) supervised by medical staff.

The AUH12O has two fully equipped intervention rooms for device implants, electrophysiological studies, and ablations, and an adjacent additional room for “simple” procedures (cardioversions, pharmacological tests, etc.). They are active for five morning shifts and three afternoon shifts per week. The procedures are performed on three types of patients: urgent hospitalized from the emergency department, hospitalized on a scheduled basis in conventional hospitalization beds, or in a day hospital (DH).

A contingency plan for the AUH12O was developed in order to meet the health care needs of patients with arrhythmias in a safer manner, based on: (1) intensification of telehealth clinics; (2) cancellation of all non-urgent ambulatory procedures in favor of those that cannot be postponed; (3) scheduling of some outpatient procedures previously performed under hospitalization; (4) design of a “COVID-free” circuit for ambulatory patients; and (5) development of specific protocols for the management of patients with COVID-19 within the electrophysiology rooms. According to these changes, the analyzed period was divided into two stages: a “pre-COVID stage” from January 12 to March 8, 2020, and a “COVID stage” from March 9 to May 2, 2020.

Data were contextualized within the pandemic situation considering the number of weekly COVID-19 infections confirmed in the region of Madrid.^3^

**Supplementary Figure 1:**
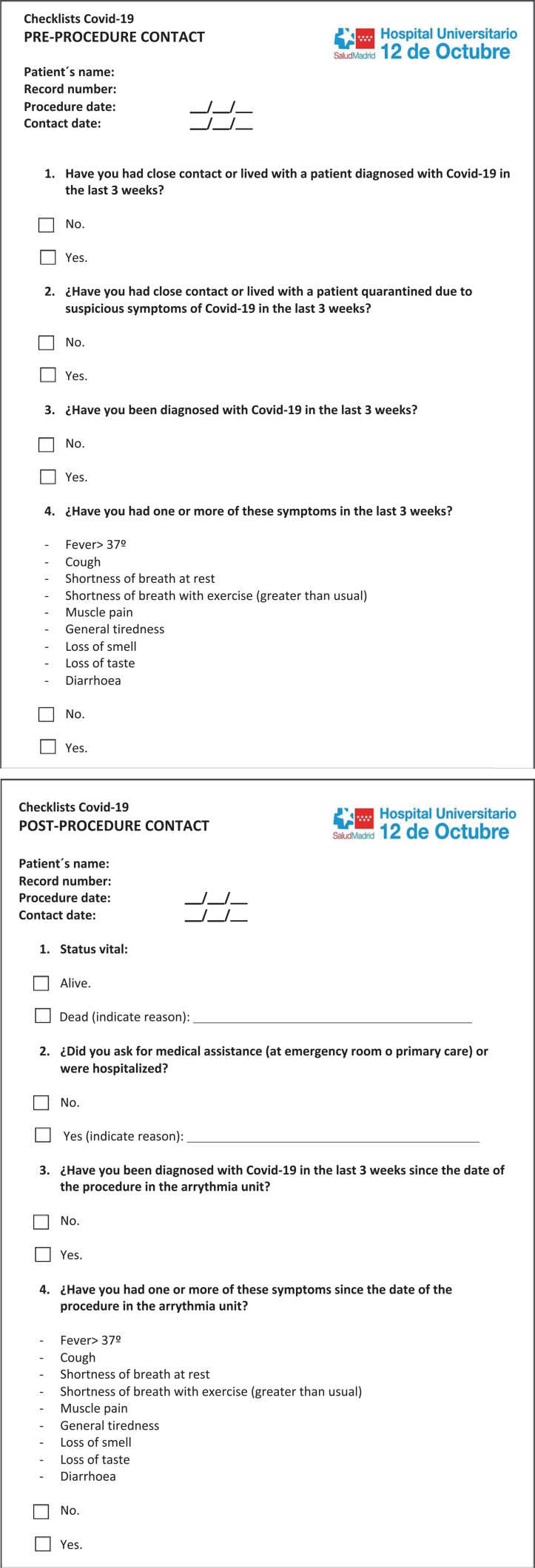
Postoperative COVID-19 follow-up questionnaire.

Finally, in the case of patients operated on in the AUH12O during the COVID stage, planned telephone follow-up was performed to determine the appearance of complications and investigate the possibility of getting COVID-19 after the procedure by means of a specific questionnaire (see **Supplementary Figure 1** online).

### Statistical analysis

Continuous variables are described as mean ± standard deviation values, and qualitative variables are described as absolute and relative frequencies (percentages). The latter were compared using the chi-squared test or Fisher’s exact test when appropriate. A two-tailed p-value was calculated, considering two-tailed p-values of less than 0.05 as statistically significant.

## Results

Various levels of adaptation of the AUH12O to the COVID-19 epidemic during the study periods are described as follows.

### Human resources

The pre-COVID stage was based on a team of four full-time electrophysiologists, two electrophysiology fellows, two residents, eight nurses, and three auxiliary nursing assistants.

During the pandemic, the workforce was reduced due to the relocation of the two fellows, the two residents, one nurse, and one nursing assistant for the direct care of COVID-19 patients. The remaining personnel (68% of the initial workforce) took over the activities assigned to the AUH12O. In addition, during the first weeks of this stage, two nurses and one nursing assistant had to take sick leave and required home isolation as a consequence of COVID-19 presumably not acquired at work. Subsequently, staffing levels gradually returned to baseline in the last weeks of this study period.

### Outpatient clinics

During the pandemic, telehealth was institutionally established as a priority with a double purpose: reducing the flow of patients throughout the hospital and promoting teleworking when possible among the employees. For this, some licenses were distributed, allowing physicians to access the electronic medical records system from their homes, enabling a virtual private network. Only those patients requiring a short physical examination or complementary test were attended to in person during the COVID stage.

A total of 3,526 consultations were attended during the analyzed period (1,792 in the pre-COVID stage and 1,734 in the COVID stage), representing a reduction of 3.2% in the latter period. All consultations were recorded in the hospital’s electronic medical records system.

**Figure 1:**
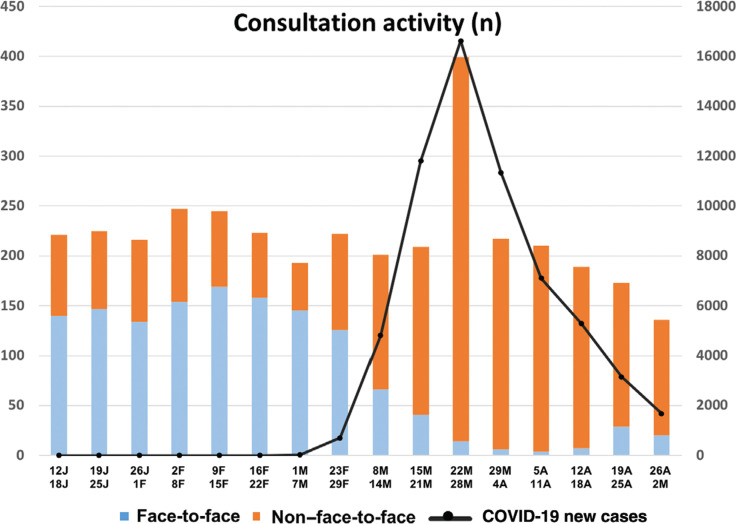
Weekly activity of the arrhythmia unit clinics (left axis) in “in person” and telehealth modalities, in the context of the epidemic situation through the number of new confirmed COVID-19 infections in the region of Madrid (right axis). Data sourced from the Carlos III Health Institute.^3^

**Figure 2:**
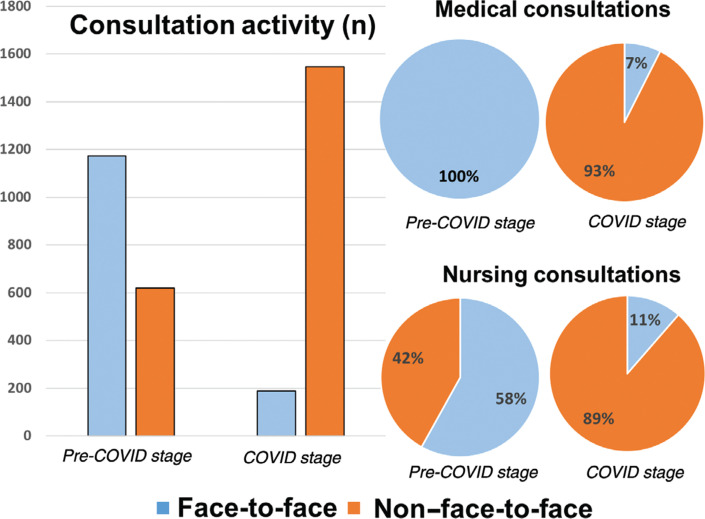
Activity of “in person” and “telehealth” clinics of the arrhythmia unit (expressed as absolute numbers and as percentages) in the previous eight weeks and during the eight weeks of the COVID-19 outbreak.

**Figures 1 and 2** show the evolution of the activity of the outpatient clinics throughout the 16 weeks. There was a 150% increase in non-contact consultations, most especially during the last week of March, coinciding with the peak of assessed community transmission of COVID-19.

As with conventional in-person consultations, test results were interpreted, electrocardiograms and reports from other centers sent via email were assessed, medication was electronically prescribed, new appointments were arranged, and complementary tests were requested when necessary. The performance of the clinics is shown in **Table 1**. During the COVID stage, patients in clinical consultations continued to be discharged although in a lesser proportion than in the pre-COVID stage (66.6% vs. 49.4%; p < 0.001) and they continued to be included in the waiting list for device-related or electrophysiology procedures in a similar percentage (15.9% vs. 13.7%; p = 0.51).

### Electrophysiology rooms’ activity

The impact of the COVID-19 epidemic in the arrhythmia intervention area during the central weeks of the COVID stage included the following measures:

- Temporary closure of one of the electrophysiology rooms for five out of the eight weeks
- Interruption of afternoon shifts for seven out of the eight weeks
- Restriction in the scheduling of hospitalizations because of the very high occupation of the hospital by patients with COVID-19 (which reached a maximum of 968 patients on April 1, 2020)
- Dedication of the interventional DH facilities as an area of support for the emergency department

This resulted in a 55.2% reduction in the interventionist activity (from 250 procedures in the pre-COVID stage to 112 in the COVID stage).

The following actions were carried out to guarantee access to invasive treatment of arrhythmias for patients who required it:

- Selection of patients in need of preferential intervention, such as device generator replacements, ventricular tachycardia, or highly symptomatic supraventricular arrhythmias
- Programming under the DH regime some ablations and device implants previously performed under standard hospitalization (given the use of the interventional DH facilities by the emergency department, a specific area for post-procedure surveillance shared with the interventional cardiology unit was set up)
- Reducing the hospital stay in case of a non-complex ablation and device implant, with systematic early discharge of the patients on the same day of the procedure
- Selection of patients at low risk of COVID-19 based on a telephone checklist based on symptoms and previous contacts **(Supplementary Figure 1)**
- Design of a clear and direct circuit to access the electrophysiology room from outside the hospital
- COVID-19 screening by obtaining a nasopharyngeal exudate for the polymerase chain reaction (PCR) test in the 24 to 72 hours prior to admission in cases requiring hospitalization

**Figure 3:**
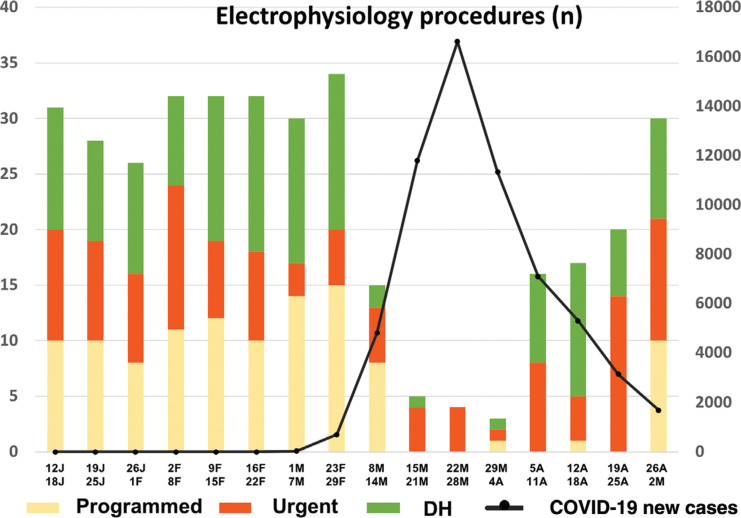
Weekly activity of the electrophysiology rooms (left axis) depending on the type of patient (urgent hospitalized, planned inpatient activity, and DH) in the context of the epidemic situation through the number of new confirmed COVID-19 infections in the region of Madrid (right axis). Data sourced from the Carlos III Health Institute.^3^

**Figure 4:**
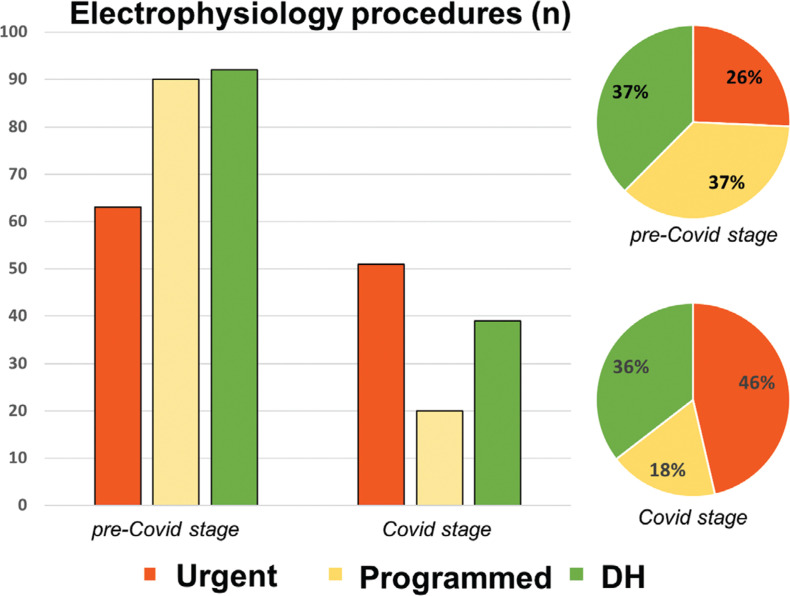
Activity of the electrophysiology rooms according to the type of patient (urgent hospitalized, planned inpatient activity, and DH) expressed as absolute numbers of procedures and as percentages, in the previous eight weeks and during the eight weeks of the COVID-19 outbreak.

**Figures 3 and 4** describe the evolution of the interventionist activity during the period of analysis. Throughout the study period, an increase in the proportion of interventions in urgent-hospitalized patients and DH patients was observed, to the detriment of those admitted for a scheduled hospitalization. Interventions under scheduled hospitalization were reactivated during the last week of the COVID stage.

**Table 2** shows the activity carried out in the electrophysiology rooms categorized by procedure and type of patient during the two periods. During the COVID stage, there was a higher proportion of pacemaker implants (14.4% vs. 25.9%; p = 0.008), whereas the rate of the remaining procedures remained stable. During the second stage, the significant increase in both procedures among urgent-hospitalized patients (25.7% vs. 46.3%; p < 0.001) and in DH procedures (17.5% vs. 30.9%; p = 0.005) was remarkable.

Fifty-five of the 59 patients (93.2%) operated during the COVID stage were successfully contacted by telephone. None of them developed COVID-19 or symptoms of the disease 25 ± 17 days after the intervention.

### Procedures performed on patients with COVID-19

**Table 3** summarizes the characteristics of the six patients with COVID-19 who underwent procedures in the electrophysiology room during the study period (11.8% of urgent procedures). Three pacemaker implants, one defibrillator implant, one recurrent intra-nodal tachycardia ablation despite drugs, and one arrhythmic storm ablation (under general anesthesia) were performed. Four of these six patients were admitted to the emergency department as a result of an arrhythmia; the remaining two were admitted for COVID-19 pneumonia and developed arrhythmias during hospitalization. All interventions took place without incident. One of the hospitalized patients with pneumonia who received a pacemaker died two weeks later from respiratory failure. The rest were discharged. The personnel involved in these procedures did not develop COVID-19 nor had suggestive symptoms of the disease.

## Discussion

The impact of the first outbreak of the COVID-19 pandemic on the health care activity of an arrhythmia unit in a university hospital in a severely affected region is described in this observational study.

The main findings of the study are as follows:

1. The epidemic led to a one-third decrease in the human resources dedicated to the AUH12O and the activity interruption of one of the two electrophysiology rooms, as well as the afternoon shifts.
2. The measures to adapt the activity of the arrhythmia units to the pandemic, established on March 9, 2020, preceded the accelerated growth phase of the pandemic in the Madrid region and the rest of Spain, where a national shutdown was declared on March 14.
3. The impact of COVID-19 on the number of consultations was minimal (reduction of 3.2%), thanks to the significant increase in non-presential consultations (by 150%). Telephone attention was efficient, allowing for treatment adjustment, patient discharge, or their inclusion on the waiting list for arrhythmia procedures.
4. The interventions in the electrophysiology rooms were reduced by 55.2%, especially due to the restriction of procedures in the first three weeks of the outbreak, when the activity was limited to hospitalized patients admitted from the emergency department.
5. The implementation of specific protocols during the pandemic allowed for the safe performance of procedures on patients with COVID-19.
6. Weeks before, the population confinement measures were reduced, and in view of the decrease in health care pressure brought about by COVID-19, it was possible to progressively resume the programmed activities and “normal” functioning in the last week of the COVID stage. There were no suspected or confirmed COVID-19 infections among patients who underwent scheduled procedures.

### In-person clinics

Our experience reveals that, in daily clinical practice, there is a chance to increase the proportion of telehealth consultations. This understanding may represent a paradigm shift in the organization of the activity of the outpatient clinics in arrhythmia units, as it provides obvious benefits in terms of costs, avoids the displacement of patients and their companions, limits the waiting times and physical occupation rates in clinics. These data suggest the need to empower telehealth for application not only in future epidemic outbreaks but also during periods of normal activity.

In our opinion, arrhythmia units can take the opportunity induced by this crisis to decisively address the challenges posed by telemedicine at multiple levels (patient education, personnel management, computer equipment, remote monitoring platforms, video calls, wearable devices, etc.) in the context of what has been called the “digital revolution in health care” as a result of the COVID-19 pandemic.^4^

### Scheduled interventions

During the study period, the need for planned interventions connected to the risk of contagion and the risk of postponing such interventions were carefully balanced. To this end, individual and epidemiological factors were considered, such as (1) the patient’s individual risk of acquiring the infection and presenting complications from COVID-19, (2) the urgency of the intervention, (3) the intensity of community transmission at each moment, and (4) the health care resources available in the hospital.

Starting from a high individual risk in most of the patients on the waiting list, efforts were concentrated on limiting the risk of infection by ensuring direct access to the electrophysiology room through a “COVID-free” circuit and minimizing their hospital stay.

Performing same-day discharge ablations and device implants is a previously described strategy that, in addition to being feasible and safe, generates patient satisfaction and reduces costs.^5–8^ The particularity of our approach to the problem was the development of a provisional DH within the area of cardiac interventionism, redistributing staff, given the occupation of the facilities of the interventional DH to support the emergency department and the extremely high occupancy of hospital beds, to where generator replacements, cardioversions, pacemaker and defibrillator implants, and certain ablations were performed.

As in the case of telehealth clinics, this experience forced us to review the absolute need for hospital admission in many of the arrhythmia procedures, especially in times of uncertainty regarding the evolution of the pandemic.

Although the definition of urgent procedures in arrhythmias can be complex, a selection was made in accordance with the previously reported document by the Heart Rhythm Society (HRS COVID-19 Task Force), which recommends postponing elective interventions in favor of those considered urgent or non-delayable.^9^

There was also controversy about when elective interventions would be resumed after the peak of the pandemic, especially after the reported increase in mortality by 20% in patients undergoing scheduled surgeries.^10^ Our group opted for an early resumption of scheduled activity, as the evidence regarding surgical interventions cannot be extrapolated to arrhythmia procedures, which are minimally invasive and require shorter hospital stays. This allowed a progressive reopening of elective activity in a safe manner during the week after following the local peak of the pandemic curve and minimizing the risk of scheduling interventions in coronavirus carriers through a telephonic epidemiological checklist **(Supplementary Figure 1)** and a prior PCR test in cases requiring hospitalization.

### Interventions in inpatients

The absolute number of urgent interventions in inpatients (hospitalized through the Emergency Department) was reduced by only 19% during the COVID stage, despite the limitations in personnel and availability of the electrophysiology rooms during the period. This decrease was less than that reported in other cardiology interventions such as percutaneous revascularization in acute myocardial infarction, whose activity in Spain was reduced by 40% during this crisis.^11^ On the other hand, we experienced a significant increase in the proportion of urgent procedures at the expense of pacemaker implants.

A recent observational study strongly suggested a higher mortality rate among patients hospitalized for COVID-19 with cardiac disease.^12^ However, whether the outcomes of electrophysiological procedures are especially worse in these patients remains unclear. Although caution on this topic is needed in the absence of other reported series, our initial experience (a positive evolution of five out of six interventions in COVID-19 patients) does not suggest that they have a particularly unfavorable prognosis in this setting, and the results are probably directly related to the course of the infection itself.

### Experiences from other centers

There are several publications by arrhythmia units proposing activity prioritization plans in electrophysiology rooms,^13^ clinical pathways for managing patients with COVID-19 (suspected or confirmed),^14^ and recommendations regarding the management of clinics and personnel restructuring.^15,16^ However, none of them provide data on the clinical results of the proposed measures. We report for the first time a comprehensive organizational experience in an arrhythmia unit during the COVID-19 outbreak, including planning, staff reordering, activity data, and clinical outcomes.

The impact of the COVID-19 outbreak on our number of electrophysiology procedures was comparable to that published in similar countries like Italy, where a survey including 84 Italian arrhythmia units showed that most of them experienced a reduction in device implantations and cardiac ablations of more than 50%.^17^

### Limitations

The main limitation of this study is that it was restricted to a single center. Although the measures implemented in the AUH12O during the pandemic are in line with the recommendations of scientific societies and the publications of other groups, they occurred in a particularly serious context, which may differ from other institutions. On the other hand, we lack an objective assessment of the performance of the actions taken in response to the epidemic, as we cannot compare our results with those of other series.

## Conclusions

The measures implemented in the AUH12O during the first outbreak of the COVID-19 pandemic in Spain helped to safely and efficiently adapt to the health care needs of patients with arrhythmias in this extraordinary situation. This experience can be useful when comparing the various strategies carried out during this period by other centers and with a look to the future planning of the arrhythmia units in future outbreaks of the pandemic. Our work may be useful when facing succeeding waves of COVID-19 in order to avoid the excess mortality indirectly caused by cardiac arrhythmias, guaranteeing the safety of the patients and the staff involved in cardiac care.

## Figures and Tables

**Table 1:** Activity of Clinics During Pre-COVID and COVID Stages

		Pre-COVID Stage (January 12–March 8, 2020)	COVID Stage (March 9–May 2, 2020)	p-value
Clinical consultations				
In person		315	20	< 0.001
Waiting list entry		50 (15.9%)	5 (25%)	0.34
Discharge		210 (66.7%)	11 (55%)	0.28
Telehealth		0	249	
Waiting list entry		0 (0%)	32 (12.8%)	1
Discharge		0 (0%)	122 (49%)	1
	Total	315	269	
Device consultations				
In person		858	167	
Waiting list entry		58 (6.8%)	9 (5.4%)	0.12
Telehealth		619	1,298	
Waiting list entry		23 (3.7%)	31 (2.4%)	0.26
	Total	1,477	1,465	

COVID: coronavirus disease.

The performance is shown as a function of the number of discharges and the number of patients included in the waiting list for arrhythmia interventions in the different modalities of clinics.

**Table 2:** Activity of the Electrophysiology Rooms During Pre-COVID and COVID Stages by Procedure and Patient Type

	Pre-COVID Stage (January 12–March 8, 2020)	COVID Stage (March 9–May 2, 2020)	p-value
Procedures			
Single-/dual-chamber pacemaker	36 (14.4%)	29 (25.9%)	0.008
Single-/dual-chamber ICD	12 (4.8%)	6 (5.4%)	0.82
CRT implant	14 (5.6%)	9 (8%)	0.37
Generator replacement	41 (16.4%)	16 (14.3%)	0.61
Lead extraction	1 (0.4%)	1 (0.9%)	0.56
Left atrial appendage occlusion	4 (1.6%)	0 (0%)	0.31
EPS/simple ablation	36 (14.4%)	18 (16.1%)	0.68
Complex ablation	15 (6%)	7 (6.2%)	0.93
Atrial fibrillation ablation	30 (12%)	8 (7.1%)	0.16
DC cardioversion	42 (16.8%)	13 (11.6%)	0.2
Drug challenge	4 (1.6%)	2 (1.8%)	0.9
ILR implant	15 (6%)	3 (2.7%)	0.18
Total procedures	250	112	
Patients			
Urgent admission COVID-19–positive	0 (0%)	6 (5.4%)	< 0.001
Urgent admission COVID-19–negative	63 (25.7%)	45 (40.9%)	0.004
Planned activity (inpatients)	90 (36.7%)	20 (18.2%)	< 0.001
DH (morning shift)	43 (17.5%)	34 (30.9%)	0.005
DH (afternoon shift)	49 (20%)	5 (4.5%)	< 0.001
Total patients	245	110	

COVID: coronavirus disease; COVID-19: coronavirus disease 2019; CRT: cardiac resynchronization therapy; DC: direct current; DH: day hospital; EPS: electrophysiological study; ICD: implantable cardioverter-defibrillator; ILR: implantable loop recorder.

**Table 3:** COVID-19 Patients Who Underwent Interventional Procedures in the Electrophysiology Room During the COVID-19 Outbreak

Patients	Age	Sex	Reason for Admission	Medical History	COVID-19	Arrhythmia	Intervention	Success
#1	90	M	Fever and dyspnea	Hypertension Stroke	Bilateral pneumonia	Second- and third-grade AVB	Single-chamber pacemaker	Yes
#2	83	M	Syncope	Hypertension. TAVI (2017)	Bilateral pneumonia	First-grade AVB and LBBB	Dual-chamber pacemaker	Yes
#3	68	F	Scheduled surgery	Duodenal cancer	Bilateral pneumonia	Incessant AVNRT	Ablation	Yes
#4	60	M	Chest pain	Previous infarction	Mild infection	VT	Single-chamber ICD	Yes
#5	86	M	Syncope	Mild cognitive impairment	Mild infection	Second-grade AVB	Dual-chamber pacemaker	Yes
#6	72	M	Chest pain	Previous infarction	Bilateral pneumonia	Arrhythmic storm	Ablation	Yes

AVB: atrioventricular block; AVNRT: atrioventricular nodal reentrant tachycardia; COVID-19, coronavirus disease 2019; F: female; ICD: implantable cardioverter-defibrillator; LBBB: left bundle branch block; M: male; TAVI: transaortic valve implantation; VT: ventricular tachycardia.

*The patient died due to respiratory failure caused by COVID-19 pneumonia.
